# Supplementary material for: Disentangling the effects of structure and lone-pair electrons in the lattice dynamics of halide perovskites
Source: Nat Commun. 2024 May 17;15:4184. doi: 10.1038/s41467-024-48581-x (PMC11101661; doi:10.1038/s41467-024-48581-x)
Supplement: Supplementary file 1 — Supplementary Information [file 41467_2024_48581_MOESM1_ESM.pdf]

Supplementary Information to: Disentangling the Effects of Structure and Lone-Pair Electrons in the Lattice Dynamics of Halide Perovskites

Sebastián Caicedo-Dávila,[1] Adi Cohen,[2] Silvia G. Motti,[3,4] Masahiko Isobe,[5] Kyle M. McCall,[6,7,*] Manuel Grumet,[1] Maksym V. Kovalenko,[6,7] Omer Yaffe,[2] Laura M. Herz,[3,8] Douglas H. Fabini,[5,9,†] and David A. Egger[1,‡]

[1] *Physics Department, TUM School of Natural Sciences, Technical University of Munich, 85748 Garching, Germany*
[2] *Department of Chemical and Biological Physics, Weizmann Institute of Science, Rehovot 76100, Israel*
[3] *Clarendon Laboratory, Department of Physics, University of Oxford, Parks Road, Oxford, OX1 3PU, United Kingdom*
[4] *School of Physics and Astronomy, Faculty of Engineering and Physical Sciences, University of Southampton, University Road, Southampton SO17 1BJ, United Kingdom*
[5] *Max Planck Institute for Solid State Research, 70569 Stuttgart, Germany*
[6] *Laboratory of Inorganic Chemistry, Department of Chemistry and Applied Biosciences, ETH Zurich, CH-8093 Zürich, Switzerland.*
[7] *Laboratory for Thin Films and Photovoltaics, EMPA - Swiss National Laboratories for Materials and Technology, CH-8600 Dübendorf, Switzerland*
[8] *TUM Institute for Advanced Study, Technische Universität München, 85748 Garching, Germany*
[9] *Department of Chemistry, Massachusetts Institute of Technology, 02139 Cambridge, MA, USA*

Supplementary Note 1: Vibronic anharmonicity and the strength of the Pseudo-Jahn−Teller effect

The strength of the pseudo-Jahn−Teller (PJT) effect differs considerably between $CsSrBr_3$ and $CsPbBr_3$, as we demonstrate here. The energies of the two-level PJT problem with linear vibronic coupling (see Bersuker Section 4.1 for background and derivation)[1] can be written as:

$$\epsilon_{\pm}(Q) = \pm\Delta + \frac{1}{2}\left(K_0 \pm F^2/\Delta\right)Q^2 \mp \frac{1}{4}\left(F^4/\Delta^3\right)Q^4 + ..., \tag{1}$$

where Q is the nuclear distortion coordinate ($Q = 0$ in the high symmetry reference case, *i.e.* octahedral coordination), K_0 is the primary force constant in the absence of the vibronic interaction, F is the off-diagonal linear vibronic coupling constant, and 2Δ is the energy separation between the electronic ground and (degenerate) excited states in the reference case. The so-called "strong" case of the PJT effect, which produces a spontaneous distortion that breaks inversion symmetry, occurs when $\Delta < F^2/K_0$. Otherwise, in the so-called "weak" case, there is no spontaneous distortion, but the effective force constant of the ungerade nuclear displacements in the ground state is nonetheless vibronically softened by an amount F^2/Δ and vibronic anharmonicity is introduced (distinct from proper anharmonicity, see Section 2.4 of Bersuker).[1] The magnitudes of these vibronic impacts on the ground state (softening of the quadratic force constant, anharmonicity) take on a continuum of values in various systems. We designed the comparison between $CsPbBr_3$ and $CsSrBr_3$ to test the influence of the vibronic anharmonicity on the lattice dynamics of halide perovskites – despite both formally exhibiting the weak PJT effect, as we show below, the vibronic contributions are markedly different in magnitude in these two systems, with $CsPbBr_3$ lying much closer to a spontaneous acentric distortion.

To parameterize this model in terms of observables from Born-Oppenheimer density functional theory (DFT), we rewrite the energy of the lower surface (electronic ground state) up to 4th order and drop the "−" subscript, yielding:

$$\epsilon(Q) + \Delta = a_2 Q^2 + a_4 Q^4, \tag{2}$$

where $a_2 = \frac{1}{2}\left(K_0 - F^2/\Delta\right)$ and $a_4 = \frac{1}{4}\left(F^4/\Delta^3\right)$. We fit a_2 and a_4 to DFT total energies from a series of distorted structures (using `VASP`, PAW potentials, PBE functional, 600 eV plane wave cutoff, 7×7×7 k-point mesh for oxide

---

* Present address: Department of Materials Science and Engineering, University of Texas at Dallas, 75080 Richardson, TX, USA
† fabini@mit.edu
‡ david.egger@tum.de

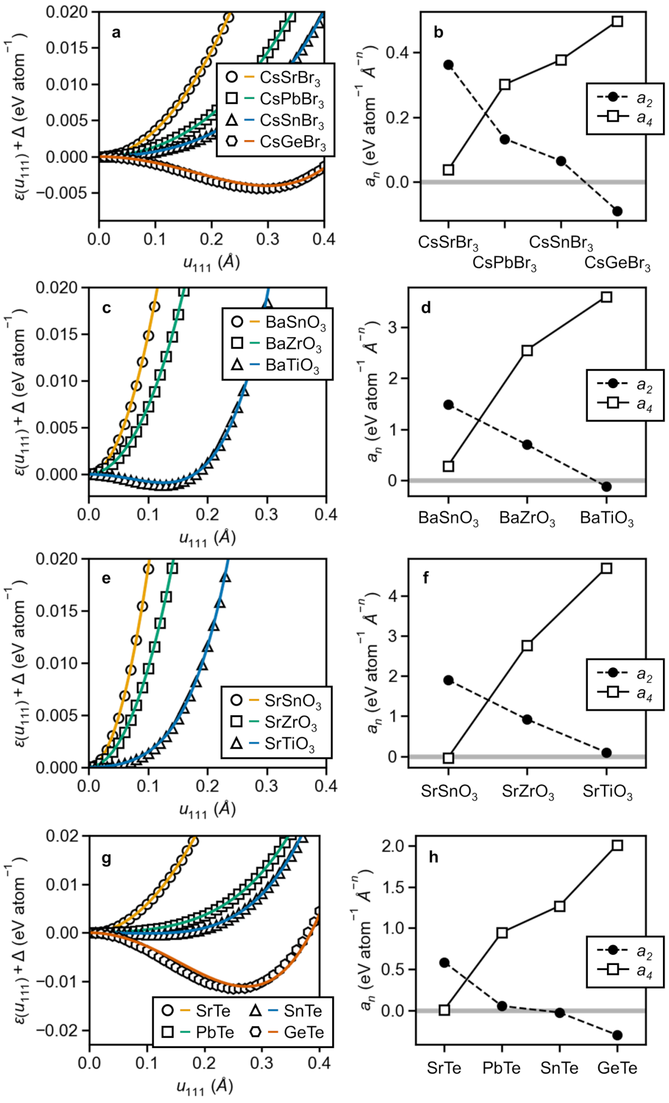

Supplementary Figure 1. **Energetics of trigonal distortions.** DFT-computed energies as a function of trigonal displacement of the octahedral cations, u_{111}, and fitted 2-level PJT model parameters for bromide perovskites (panels a and b), oxide perovskites (panels c-f), and rocksalt tellurides (panels g and h). In panels a, c, e, and f, markers are data and solid lines are fits to Equation 2. Fitted values of a_2 and a_4 are visualized panels b, d, f, and h across each chemical series. For each family of compounds except the Sr-oxides, a progression from negligible to weak to strong PJT effects is found as a_4 increases and a_2 decreases. For the Sr-oxides, the progression is similar but a_2 does not quite cross 0 for paraelectric $SrTiO_3$, unlike for ferroelectric $BaTiO_3$, illustrating the secondary influence of chemical pressure. A strong PJT effect ($a_2 < 0$) is found for systems with acentric cation site symmetry ($3m$) in the experimental ground state crystal structures ($CsGeBr_3$, $BaTiO_3$, SnTe, GeTe, all space group $R3m$). $CsSnBr_3$, recently shown to exhibit acentric Sn site symmetry (1, space group $P2_1$) at low temperatures[2] lies near $a_2 = 0$, with a weak PJT erroneously predicted at the equilibrium unit cell size. All other cases with $a_2 > 0$ have cations on sites with inversion symmetry in the experimental ground state crystal structures ($m\bar{3}m$ for $BaSnO_3$, $BaZrO_3$, SrTe, and PbTe; $\bar{1}$ for $CsSrBr_3$, $CsPbBr_3$, $SrSnO_3$, and $SrZrO_3$; $4/m$ for $SrTiO_3$). $CsSrBr_3$, (Ba,Sr)SnO_3, and SrTe exhibit negligible PJT effects: the vibronic anharmonicity coefficient, a_4, is much smaller than for the other compounds in each series.

and bromide perovskites, 9×9×9 for rocksalt tellurides). It is not straightforward to extract the appropriate Δ from DFT so that F and K_0 can be obtained explicitly from a_2 and a_4. Nevertheless, there is insight to be had in the values of a_2 and a_4 directly: $a_2 < 0$ corresponds to the strong case of the PJT effect, and $a_4 \to 0$ as the PJT effect becomes negligible (*i.e.*, either as the vibronic coupling constant $F \to 0$ or as the energy separation between the ground state and the degenerate excited states $2\Delta \to \infty$). Despite the differing symmetries of the degenerate excited state orbitals for d^0 and s^2 cations in octahedral coordination, these two cases are known to give rise to the same, trigonal distortion.[3, 4] For this study of chemical trends and in a manner similar to previous reports,[5, 6] we apply rigid

trigonal displacements of the octahedral cations along [111] rather than perturbing along the exact phonon eigenvector.

We examine three series of compounds (bromide perovskites, oxide perovskites with either Ba^{2+} or Sr^{2+} on the A-site, and rocksalt tellurides) which modulate the PJT effect strength across a wide range, and correlate the results (Supplementary Figures 1 and 2) to chemistry and bonding. Supplementary Figure 1 shows that, as we traverse the series $CsSrBr_3 \to CsPbBr_3 \to CsSnBr_3 \to CsGeBr_3$, the quadratic coefficient in the 2-level PJT model, a_2, softens considerably and eventually becomes negative, corresponding to the strong case of the PJT effect and the formation of lone pairs in $CsGeBr_3$. Though atomic masses play a role via the primary force constants, K_0, one can see this is not the dominant factor in the trend in a_2: the vibronic contribution to the quadratic coefficient is essential. Simultaneously, the quartic coefficient, a_4, (vibronic anharmonicity) rises considerably across the series. The value of a_4 for $CsPbBr_3$ is 8 times that for $CsSrBr_3$. Thus, these two compounds are in very different PJT regimes and it is the impact of this distinction on the lattice dynamics of the two which we also probe in this study. We refer to $CsSrBr_3$ as exhibiting a "negligible" PJT effect, and contrast its behavior with that of $CsPbBr_3$, which shares a weak PJT effect with other technologically-relevant Pb(II) bromides and iodides, and with that of $CsGeBr_3$,[7] which exhibits a strong PJT effect.

Supplementary Figure 1 reveals similar trends for the oxide perovskites and the rocksalt tellurides, with perfect correspondence between the finding of a strong PJT effect ($a_2 < 0$) and those systems which exhibit acentric cation environments in their ground state phases. Notably, the strengths of the PJT effects we fit numerically also perfectly track the chemical trends expected for d^0 cations (*e.g.* Ti^{4+} exists in more strongly distorted environments than Zr^{4+})[8] and s^2 cations (*e.g.* distortions increase in the order $Pb^{2+} < Sn^{2+} < Ge^{2+}$).[9]

Supplementary Figure 2 places the fitted PJT model parameters onto a unified scale to show how they vary between the chemical families. Fixed-Δ, varying-F curves of $(a_2(K_0, F, \Delta), a_4(F, \Delta))$ are shown for evenly spaced values of K_0 to illustrate that the families are primarily separated by their varying elastic properties in the absence of vibronic coupling (K_0), while intra-family variation can be roughly explained by differing vibronic coupling coefficients, F. Fixed-F, varying-Δ curves have a different functional form and fit the intra-family variation less well, suggesting the energy separation between the ground and degenerate excited states plays a secondary role in modulating the PJT effect strength across these particular series.

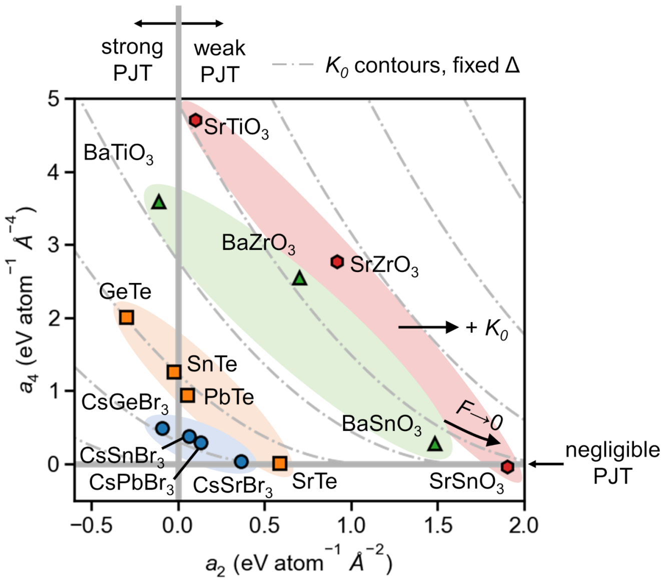

Supplementary Figure 2. **Modulating the PJT effect.** Values of a_2 and a_4 from fitting DFT energies to Equation 2 across several bromide perovskites, oxide perovskites, and rocksalt tellurides. To illustrate the influences of the primary force constant, K_0, and vibronic coupling constant, F, contours of K_0 at a fixed Δ are shown. Each family of materials falls roughly onto a curve of varying F, with the families differentiated from one another by different elastic properties (K_0). Notably, the series of bromide perovskites runs from a strong PJT effect ($CsGeBr_3$) through a weak PJT effect ($CsPbBr_3$) to a negligible PJT effect ($CsSrBr_3$) as the vibronic coupling coefficient, F, decreases towards zero. Additionally, the trends in a_2 show that softening of the harmonic force constant is not merely a mass effect, but includes a substantial vibronic contribution to the curvature.[1]

Supplementary Note 2: Crystal growth and processing of high-quality $CsPbBr_3$

Synthesis and Purification Runs: 6.423 g of CsBr (ChemCraft, 99.999%) and 11.077 g of $PbBr_2$ (Sigma Aldrich, 99.999%) were mixed and ground together thoroughly using a mortar under Ar environment. This material was then flame-sealed under 1.4×10^{-2} mbar vacuum into a fused silica ampoule (i.d. 10 mm) with a sharp tip. The ampoule was placed in the hot zone of a custom-built 3-zone Bridgman furnace (HTM Reetz). The temperatures were set to 675, 400 and 400 °C. The sample was left overnight to ensure a full melt and synthesis reaction, then moved through the furnace at a speed of 4.86 mm/h while undergoing 0.3 rpm rotation until it had passed outside the furnace. The sample was reset and the same temperature profile applied, and moved through the furnace more slowly (2.52 mm/h) to fully segregate black impurities near the top of the ingot. The resulting ingot was opened under Ar environment and the black regions at the top were cut off and discarded. The material was then broken into chunks to reduce the risk of thermal expansion cracking the ampoule and flame-sealed under 1.1×10^{-2} mbar vacuum into a new fused silica ampoule (i.d. 10 mm) with a sharp tip. A final purification run, with identical conditions to the previous run showed no further black impurities. The material was then sufficiently pure to yield higher crystallinity.

Crystal Processing: The ingot was opened under Ar environment and cut into 2 mm-thick wafers using a Crystal Systems Corporation Cu-02 Desktop Crystal Cutter with Goniometer operating at 60 rpm with oil-based lubricant. The surfaces of these wafers were polished using a Crystal Systems Corporation TP-02 Polisher operating at 20 rpm, with MicroMesh SiC cutting papers used to get successively finer surfaces with a final polish of 12 000 grit producing an optical mirror-like surface. These processing steps were completed under Ar environment to preserve the pristine surfaces and the crystals were sealed under Ar for transport, ensuring that both the raw material and as-grown crystals were never exposed to ambient conditions.

Supplementary Note 3: Lattice parameters and volumetric expansion coefficient

Supplementary Table 1 shows the theoretical lattice parameters, obtained from fitting the free energy as a function of volume to a Birch-Murnaghan equation of state[10, 11] for $CsPbBr_3$ and $CsSrBr_3$ in the cubic $Pm\bar{3}m$ and orthorhombic $Pbnm$ phases. The details of the density-functional theory (DFT) calculations are given in the Methods section of the main text. The experimental lattice parameters estimated from X-ray diffraction (XRD) experiments at ≈527 K and ≈308 K for the cubic and orthorhombic phases, respectively, are provided as well. Notably, the difference between theoretical and experimental lattice constants is lower than 1% for both phases.

Supplementary Table 1. DFT-calculated lattice constants of the cubic $Pm\bar{3}m$ and orthorhombic $Pbnm$ phases of $CsPbBr_3$ and $CsSrBr_3$. In parenthesis: experimental lattice parameters measured at 527 K ($Pm\bar{3}m$) and 308 K ($Pbnm$).

Compound	Cubic	Orthorhombic		
	a	a	b	c
$CsPbBr_3$	5.81 (5.88)	8.20 (8.22)	8.25 (8.27)	11.75 (11.78)
$CsSrBr_3$	5.92 (5.93)	8.33 (8.34)	8.28 (8.25)	11.86 (11.83)

Supplementary Figure 3 shows the cell volume per formula unit as a function of temperature, calculated from the lattice parameters measured in XRD experiments, which are compared to literature data[12] of $CsSrBr_3$:Eu in Supplementary Figure 4. Supplementary Table 3 shows the DFT-optimized structure of $CsSrBr_3$ in the $Pnma$ phase. We fitted a second order polynomial model for the volume:

$$V[\text{Å}^3] = a + b * T[\text{K}] + c * T^2[\text{K}] \tag{3}$$

The fitted coefficients for $CsPbBr_3$ and $CsSrBr_3$ are shown in Supplementary Table 2, and the resulting models are shown as dashed lines in Supplementary Figure 3a. The volumetric thermal expansion coefficient can be defined as:

$$\alpha_V \ [\text{K}^{-1}] = \frac{1}{V}\frac{\partial V}{\partial T} \tag{4}$$

We used Equation 4 to numerically estimate by central differences the values of α_V from the volumes measured in XRD at each temperature. The result is shown in the scatter plots of Supplementary Figures 3b and c for $CsPbBr_3$ and $CsSrBr_3$, respectively. Furthermore, $\alpha_V(T)$ can be estimated in another way by using the model in Equation 3 in 4. The result of this procedure is shown as dashed lines in Supplementary Figures 3b and c for $CsPbBr_3$ and $CsSrBr_3$, respectively. These models describe the experimental data in a satisfactory manner. Specifically, the coefficients α_V of $CsPbBr_3$ and $CsSrBr_3$ were estimated to be $\approx 1.29 \times 10^{-4}$ K^{-1} and $\approx 1.32 \times 10^{-4}$ K^{-1} at 300 K, respectively, in excellent agreement with values reported in the literature.[12] As temperature increases, α_V slightly decreases to $\approx 0.76 \times 10^{-4}$ K^{-1} and $\approx 0.78 \times 10^{-4}$ K^{-1} for $CsPbBr_3$ and $CsSrBr_3$ at 700 K, respectively.

Supplementary Table 2. Coefficients fitted to the data of volume (per formula unit) as a function of temperature. These coefficient determine the model of thermal expansion in $CsPbBr_3$ and $CsSrBr_3$.

Coefficient	$CsPbBr_3$	$CsSrBr_3$
a [Å^3]	1.913×10^2	1.939×10^2
b [Å^{3}K^{-1}]	3.339×10^{-2}	3.446×10^{-2}
c [Å^{3}K^{-2}]	-1.245×10^{-5}	-1.281×10^{-5}

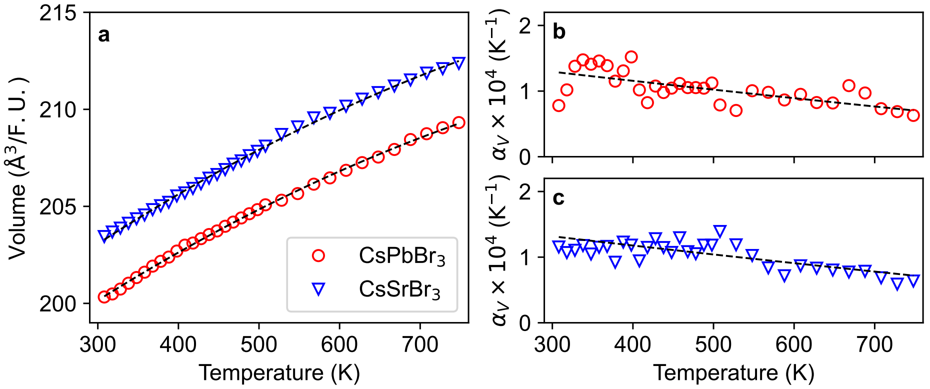

Supplementary Figure 3. **Thermal expansion.** a) Unit cell volume (per formula unit) as a function of temperature for $CsPbBr_3$ and $CsSrBr_3$. The corresponding volumetric thermal expansion coefficient for b) $CsPbBr_3$ and c) $CsSrBr_3$. The dashed black lines correspond to the values modeled with a second order polynomial (see text for details).

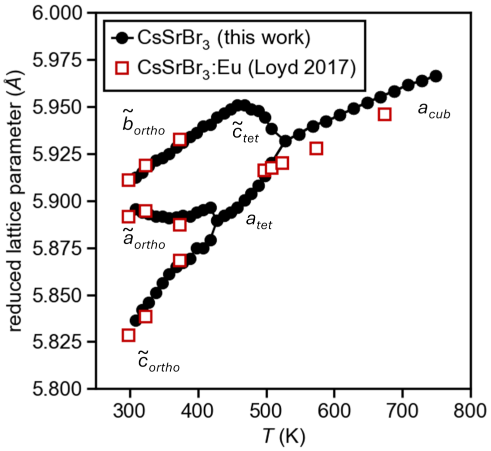

Supplementary Figure 4. **Comparison of temperature-dependent lattice parameters.** Comparison of reduced lattice parameters from powder diffraction for $CsSrBr_3$ (this work) and $CsSrBr_3$:Eu,[12] with the orthorhombic phase expressed in the *Pnma* setting. Agreement is favorable, with a possible small discrepancy in transition temperatures and cubic phase lattice parameter.

Supplementary Note 4: Infrared spectra and fitting details

Due to the large LO/TO splitting and anharmonic phonon coupling in halide perovskites (HaPs), we used a four parameter semi-quantum (FPSQ) model to fit the measured infrared (IR) reflectivity spectra. In this model, proposed by Berreman and Unterwald,[13] the dielectric function is not restricted to the harmonic characteristics of an additive Lorentz dispersion, and compared to the latter, it allows for a different frequency ($\omega_{\mathrm{LO/TO}}$) and damping ($\gamma_{\mathrm{LO/TO}}$) of the LO and TO phonons. This model has been successfully used to describe the IR response of halide and oxide perovskites, as well as materials with Reststrahlen and anharmonicity signatures.[14–17] Within the FPSQ model, the complex dielectric function can be written as:

$$\varepsilon(\omega) = \varepsilon_\infty \prod_{j=1}^{N_{\mathrm{osc}}} \frac{\omega_{\mathrm{LO},j}^2 - \omega^2 + i\omega\gamma_{\mathrm{LO},j}}{\omega_{\mathrm{TO},j}^2 - \omega^2 + i\omega\gamma_{\mathrm{TO},j}}, \tag{5}$$

where ω is the frequency of incident light, N_{osc} is the number of polar phonons and ε_∞ is the high-frequency dielectric constant, which we fix to the values calculated using density-functional perturbation theory (DFPT) for the orthorhombic phases ($\varepsilon_\infty = 4.3$ and 2.9 for $CsPbBr_3$ and $CsSrBr_3$, respectively). The reflectivity at quasi-normal

Supplementary Table 3. Fractional coordinates of DFT-optimized $CsSrBr_3$ in the *Pnma* phase with lattice constants $(8.281, 11.797, 8.234)$. The coordinates are reported per element proceeding along Cs (4 atoms), Sr (4 atoms), and Br (12 atoms).

0.0782794124058532	0.7500000000000000	0.0237544762301769
0.9217205875941468	0.2500000000000000	0.9762455237698231
0.4217205875941468	0.2500000000000000	0.5237544762301769
0.5782794124058532	0.7500000000000000	0.4762455237698231
0.5000000000000000	0.5000000000000000	0.0000000000000000
0.0000000000000000	0.5000000000000000	0.5000000000000000
0.5000000000000000	0.0000000000000000	0.0000000000000000
0.0000000000000000	0.0000000000000000	0.5000000000000000
0.6952342785779840	0.4539374361406396	0.6946749577520503
0.3047657214220160	0.5460625638593604	0.3053250422479497
0.8047657214220160	0.5460625638593604	0.1946749577520503
0.1952342785779839	0.4539374361406396	0.8053250422479497
0.3047657214220160	0.9539374361406396	0.3053250422479497
0.6952342785779840	0.0460625638593604	0.6946749577520503
0.1952342785779839	0.0460625638593604	0.8053250422479497
0.8047657214220160	0.9539374361406396	0.1946749577520503
0.4936690208802648	0.7500000000000000	0.9074751120548148
0.5063309791197352	0.2500000000000000	0.0925248879451852
0.0063309791197352	0.2500000000000000	0.4074751120548148
0.9936690208802648	0.7500000000000000	0.5925248879451852

incidence is described by Fresnel formula:[18]

$$R = \left| \frac{\sqrt{\varepsilon(\omega)} - 1}{\sqrt{\varepsilon(\omega)} + 1} \right|^2 \tag{6}$$

Supplementary Figures 5a and b show the measured IR reflectivity spectra of $CsPbBr_3$ and $CsSrBr_3$ in the full measurement range up to $350\,\mathrm{cm}^{-1}$, as well as the fitted FPSQ model (Equations 5 and 6). The reflectivity of $CsPbBr_3$ was fitted using 4 and 7 modes for $CsPbBr_3$ and $CsSrBr_3$, respectively. Further increasing N_{osc} did not improve the fitting quality ($\chi^2 = 0.991$ and 0.999 for $CsPbBr_3$ and $CsSrBr_3$, respectively), while increasing the computation effort.

The TO modes appear as poles of the dielectric function, while the LO modes as zeros. Thus, the imaginary part of the complex dielectric function (ε'') gives the positions of the TO modes and the damping function of the TO, *i.e.*, $\mathrm{Im}(-1/\varepsilon(\omega))$, gives the position of the LO modes.[19, 20] Supplementary Figures 5c and d show the values of $\varepsilon''(\omega)$ and $\mathrm{Im}(-1/\varepsilon(\omega))$ as obtained for $CsPbBr_3$ and $CsSrBr_3$, respectively. The position of the peaks confirm the softening of the dominant TO mode, as well as the enhanced LO/TO splitting, of $CsPbBr_3$ compared to $CsSrBr_3$, as discussed in the main text. Furthermore, the shape of $\varepsilon''(\omega)$ is in remarkable agreement to the DFT-based IR activities calculated in the main text, although a systematic error in the frequencies of the TO modes remains noticeable. This allows us to assign the dominant TO modes of $CsPbBr_3$ and $CsSrBr_3$, at 99.7 and $163.9\,\mathrm{cm}^{-1}$, respectively, to the $B_{3\mathrm{u}}$ irreducible representation.

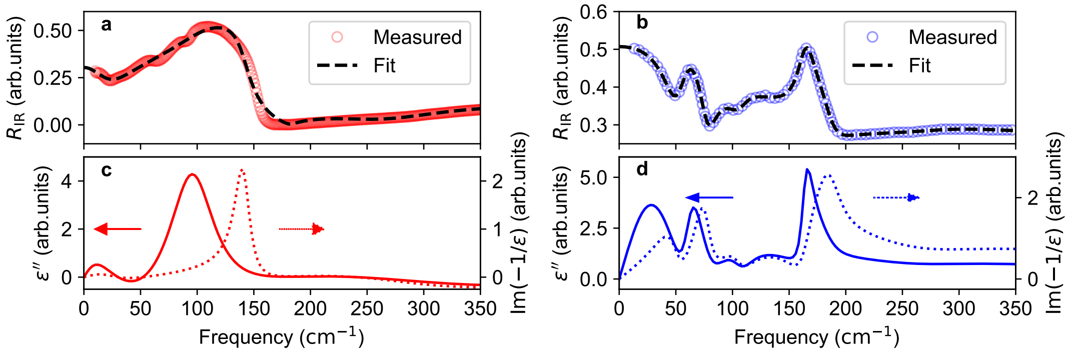

Supplementary Figure 5. **Complete IR data for orthorhombic phases.** IR-reflectivity spectra and FPSQ model fitting of a) $CsPbBr_3$ and b) $CsSrBr_3$ measured at room temperature, showing the full range of frequencies ($0\,cm^{-1}$ to $350\,cm^{-1}$). No features were detected above $\approx 200\,cm^{-1}$ The imaginary part of the dielectric function $\varepsilon''(\omega)$ and the damping function $Im(-1/\varepsilon(\omega))$ (dotted curves) of $CsPbBr_3$ and $CsSrBr_3$ are shown in panels c) and d), respectively.

Supplementary Note 5: Temperature-dependent Raman spectra of $CsSrBr_3$

Supplementary Figure 6 shows temperature-dependent Raman spectra of $CsSrBr_3$. Supplementary Figure 7 reports results from a control experiment showing that the main Raman feature of quartz is not visible in the Raman spectrum of $CsSrBr_3$.

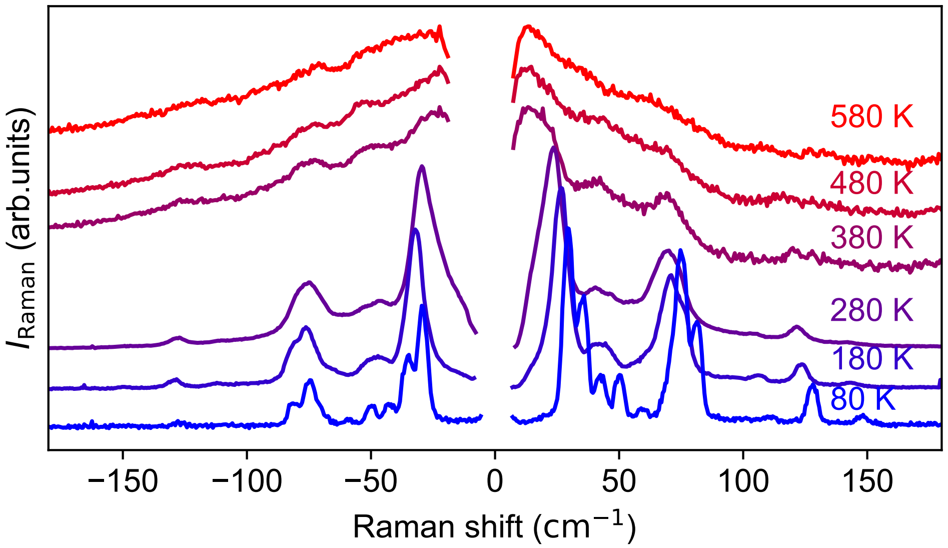

Supplementary Figure 6. **Temperature-dependent Raman spectra of $CsSrBr_3$.** Raman spectra from 80 K (blue) to 580 K (red) in steps of 100 K. No abrupt change is observed at the phase transition around 520 K. The peaks' energy redshifts with increasing temperature, and a central peak is merged at high temperature, indicating a dynamic symmetry breaking of the cubic phase, similar to $CsPbBr_3$.

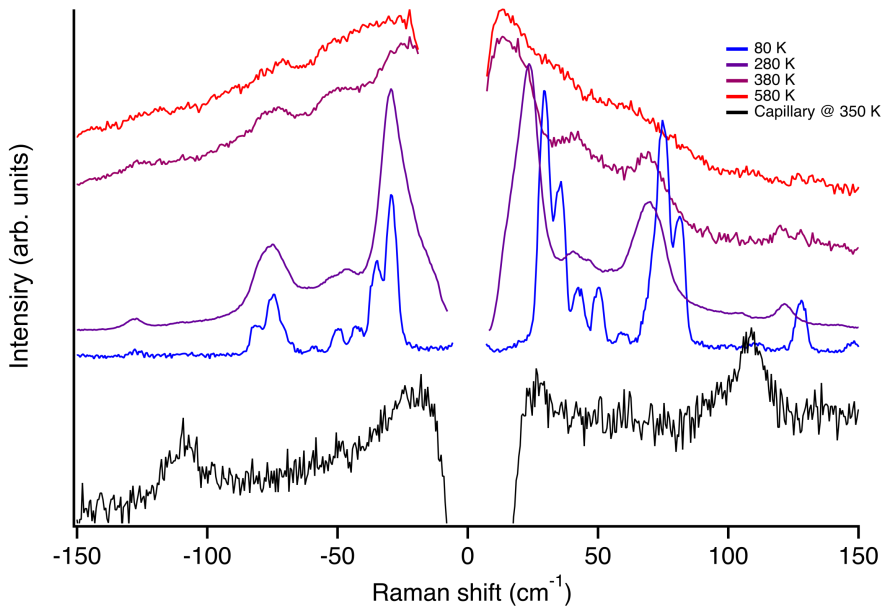

Supplementary Figure 7. **Control experiment for quartz.** Raman spectrum of quartz capillary compared to the Raman spectra of $CsSrBr_3$ at various temperatures. The main feature in the Raman spectrum of quartz at $\sim$110 cm^{-1} is not visible in the spectra of $CsSrBr_3$.

Supplementary Note 6: Calculation of the Raman and IR activities

We calculated the Raman activity tensors, $\mathbf{I}_{\text{Raman}}$, and IR activities, I_{IR}, using the `Phonopy-Spectroscopy` package[21, 22]. $\mathbf{I}_{\text{Raman}}$ can be calculated from the derivative of the polarizability tensor with respect to the phonon modes eigenvectors:[23, 24]

$$\mathbf{I}_{\text{Raman}}(s) \propto \frac{\partial \boldsymbol{\alpha}}{\partial Q(s)} \equiv \frac{\partial \boldsymbol{\epsilon}^{\infty}}{\partial Q(s)}, \tag{7}$$

where s is the phonon mode index, $Q(s)$ is the normal mode coordinate, $\boldsymbol{\alpha}$ is the polarizability tensor, and $\boldsymbol{\epsilon}^{\infty}$ is the macroscopic high-frequency dielectric tensor, which we calculated using DFPT, as implemented in `VASP`.[25] The scalar Raman intensities, I_{Raman}, are calculated from the components of the tensor, averaged for the geometry, as detailed in Refs. 23 and 21. The calculated I_{Raman} are broadened with Lorentzian functions of using a width of 5 cm^{-1}, so that the modes can be distinguished (see Figure 3, main text).

The IR spectra were calculated from $I_{\text{IR}}(s)$ for the phonon modes at the center of the Brillouin zone (BZ) as follows. The value for I_{IR} is equal to the derivative of the macroscopic polarization, $\mathbf{P}$, with respect to the displacements along the phonon-mode coordinates. The calculation is simplified by using the Born effective charges, $Z^{*j}_{\alpha,\beta}$, and the Γ-point displacements of the phonon modes, $X^{j}_{\beta}(s)$, (*i.e.*, eigenvectors normalized by the square root of the atomic masses) as detailed elsewhere:[21, 23]

$$I_{\text{IR}}(s) = \sum_{\alpha=1}^{3} \left| \frac{\partial P_{\alpha}}{\partial Q(s)} \right|^2 = \sum_{\alpha=1}^{3} \left| \sum_{j=1}^{N_{\text{atoms}}} \sum_{\beta=1}^{3} Z^{*j}_{\alpha,\beta} X^{j}_{\beta}(s) \right|^2, \tag{8}$$

where α and β are indices of Cartesian directions and j is the atomic index. Equation 8 simplifies the calculation of IR spectrum, since the values for $\boldsymbol{Z}^{*j}$ can be obtained from a single DFPT calculation.

Supplementary Note 7: Ion mass and bonding effect on IR spectra of $CsPbBr_3$ and $CsSrBr_3$

In the main text, we discussed a large blue shift of the IR spectrum of $CsSrBr_3$ with respect to $CsPbBr_3$. The blue shift of the Raman spectrum is considerably smaller ($\approx$4 cm^{-1}, an order of magnitude smaller than the shift of the IR

spectrum). To understand the role of the ionic mass in the frequency shift, we performed a thought experiment in the calculation of the IR and Raman spectra of $CsSrBr_3$. We calculated the normal modes of $CsSrBr_3$ using a fictitious mass of Sr that is equal to the mass of Pb, *i.e.*, $M_{Sr^*} = M_{Pb}$. Since IR and Raman only probe phonon modes in the center of the BZ, we focus and show only the modes at the Γ-point. Furthermore, we used the displacements of phonon modes in the fictitious system, $X_\beta^{Sr^*}$, to calculate the IR and Raman activities as in Equations 7 and 8 (see Supplementary Figure 8b and c). Supplementary Figure 8a shows the phonon density of states (DOS) projected onto the Cs, Br and M-site of $CsPbBr_3$, $CsSrBr_3$ and the fictitious $CsSr^*Br_3$. Interestingly, the PDOS of the fictitious system on the M-site recovers many features of the PDOS calculated for $CsPbBr_3$for modes with frequencies below ≈100 cm^{-1}. However, although the most prominent IR-active mode at ≈146 cm^{-1} shifts to ≈129.6 cm^{-1} in the ficticious system, it still lies considerably above the IR-active modes of $CsPbBr_3$ (≈68 cm^{-1}). Notably, mostly Br and the M-site contribute to these modes, correspond to the same irreducible representation, B_{3u}, and have very similar eigenvectors, as shown in Supplementary Figures 9a and b.
In a harmonic description of the vibrations, the interatomic force constant, k, is the other factor influencing the mode frequencies besides the atomic mass. k is obtained as the second derivative of the crystal potential energy E_{elec} (from DFT calculations) with respect to atomic displacements:[26, 27]

$$k_{\alpha,\beta}(lj, l'j') = \frac{\partial^2 E_{elec}}{\partial d_\alpha^{lj} \partial d_\beta^{l'j'}} \tag{9}$$

where d are atomic displacements and l labels the unit cell. Since E_{elec} depends on the electronic structure and chemical bonding, inspecting k serves as a proxy to analyze the impact of the lone-pair of electrons (LPE) on the frequency shift. Supplementary Figure 9c shows the normalized values of k as a function of the interatomic distance. Notably, the M–Br force constants of $CsSrBr_3$ are considerably larger than those of $CsPbBr_3$ at small interatomic distances that are close to the M–Br bond lengths. This suggests that the electronic structure, and consequently the LPE, have a dominant impact on the IR spectrum of $CsPbBr_3$ and $CsSrBr_3$. We note that at larger interatomic distances, the force constants of $CsPbBr_3$ are larger than those of $CsSrBr_3$. This reflects the long-range effects of the LPE in $CsPbBr_3$, as discussed elsewhere (see below).[28–30]

Calculations of $CsSr^*Br_3$ also show that the impact of changing the ionic mass is smaller on the Raman than on the IR spectrum (see Supplementary Figure 8c). This explains the very similar spectra measured for $CsPbBr_3$ and $CsSrBr_3$, and can be understood from the phonon PDOS at the Γ point (see Supplementary Figure 8a), which shows similarly low contributions of the M-site in both compounds at frequencies below ≈100 cm^{-1} where most of the Raman-active modes appear.

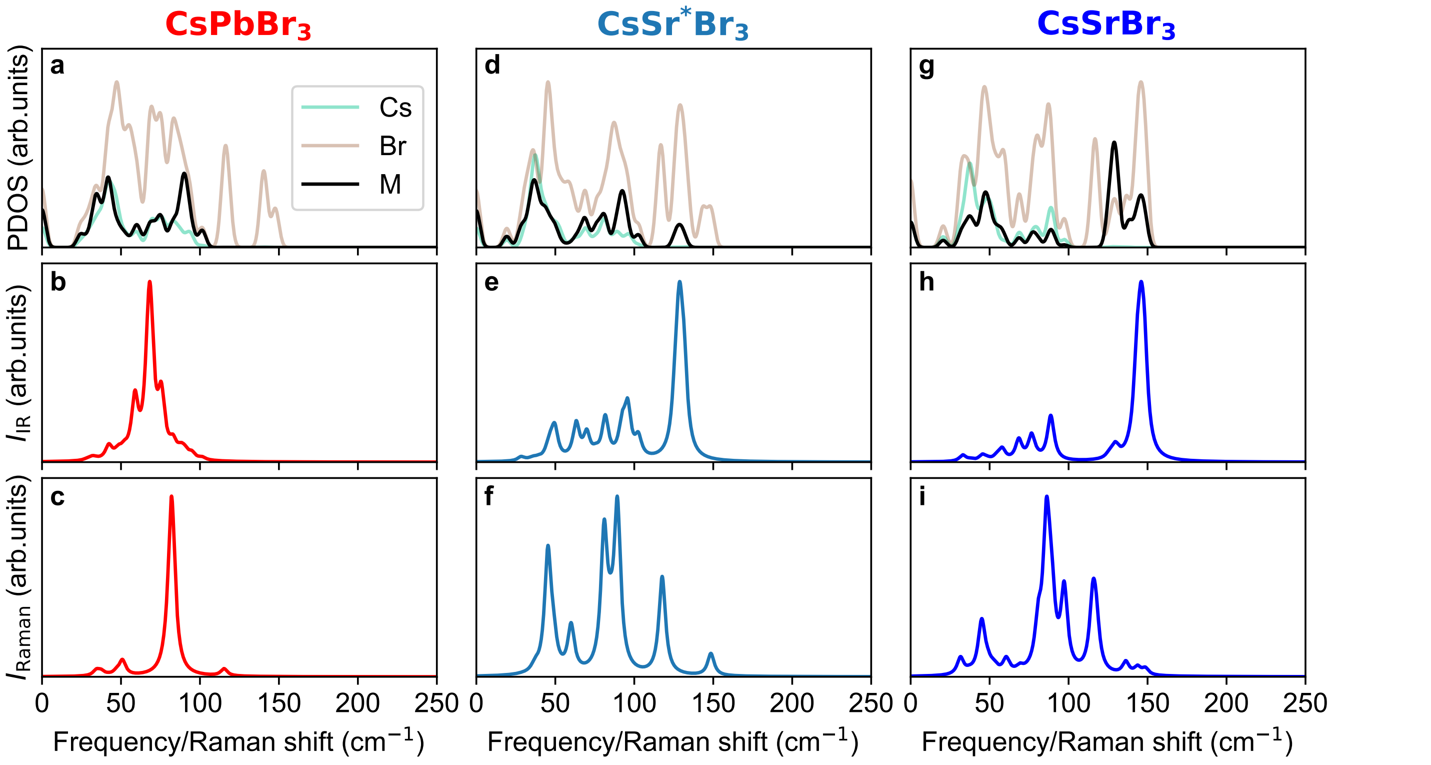

Supplementary Figure 8. **Projected phonon DOS.** Site-projected Phonon DOS at the Γ point, IR and Raman activities of orthorhombic $CsPbBr_3$ (panels a-c), $CsSr^*Br_3$ (panels d-f), and $CsSrBr_3$ (panels g-i). $CsSr^*Br_3$ is a fictitious system with modified Sr mass set as being equal to that of Pb.

Supplementary Note 8: Further analysis of molecular dynamics trajectories

It can be shown that the vibrational density of states (VDOS) equals the phonon DOS when the derivatives of the atomic coordinates (velocities, $\mathbf{v}(t)$) are given in terms of the (mass-weighted) normal mode vibrations , *i.e.*, $m_j\mathbf{v}_j(t) = m_j\dot{\mathbf{r}}_j(t) = \sum_s -i\omega_s\, Q^j(s)e^{-i\omega_s t}$.[26, 31–33] We can then write the VDOS as the power spectrum of the mass-weighted velocity autocorrelation function (VACF):

$$\mathrm{VDOS} = \int_0^\infty \frac{\sum_{j=0}^{N_{\mathrm{ions}}} \langle m_j\mathbf{v}_j(t)\cdot m_j\mathbf{v}_j(0)\rangle}{\sum_{j=0}^{N_{\mathrm{ions}}} \langle |m_j\mathbf{v}_j(0)|^2\rangle} e^{-i\omega t} dt, \tag{10}$$

where $\langle\cdot\rangle$ means the average over all time steps (i.e., shifting reference $t = 0$). Supplementary Figure 10 shows the VDOS calculated from the molecular dynamics (MD) trajectories of $CsPbBr_3$ and $CsSrBr_3$ at 525 and 570 K, respectively. Notably, the VDOS of both compounds reproduces well the large phonon DOS at low frequencies ($\leq$50 cm^{-1}) seen in the harmonic calculations. For higher frequencies, we see a strong softening of the optical modes in both $CsPbBr_3$ and $CsSrBr_3$compared to the harmonic calculations. This phonon softening has been previously discussed as a fingerprint of anharmonicity, not only for HaPs, but also for thermoelectric materials.[29, 34–37]

Supplementary Figure 11 shows the time evolution of the rotation angle around the z Cartesian axis for all eight MBr_6 octahedra of $CsPbBr_3$ and $CsSrBr_3$, calculated from the MD trajectories at 525 and 570 K, respectively. For both materials, $\phi_z(t)$ shows similar behavior. It rapidly oscillates for some tens of ps around a finite angle, $\phi_z(t) \neq 0$, before changing sign. These fast oscillations occur around distorted structures that do not exhibit the average cubic symmetry of the high-temperature HaPs, and can allow for a finite Raman response without breaking selection rules. Furthermore, the systems oscillate among these distorted structures at a much lower frequency. This kind of low-frequency dynamic disorder is present in both $CsPbBr_3$ and $CsSrBr_3$. However, it seems to be slower for the former, which results in a sharper $\Phi_z(\omega)$ towards 0 cm^{-1} for $CsPbBr_3$ (*cf.* Figure 11 and Figure 6, main text).

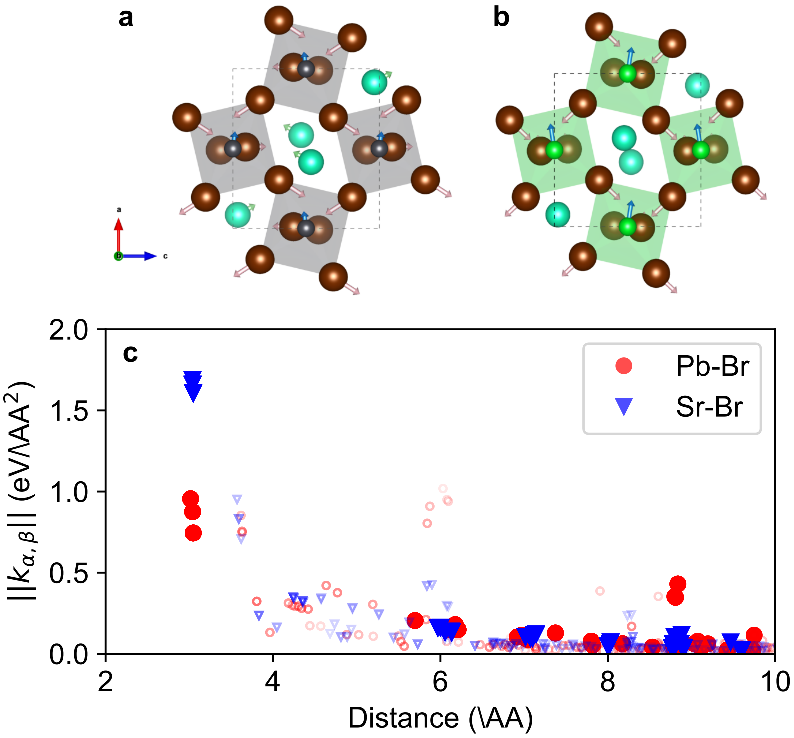

Supplementary Figure 9. **Phonon eigenmodes and interatomic force constants.** Schematic representation of the phonon-mode eigenvector involved in the strongest IR-active phonon mode with irreducible representation B_{3u} of orthorhombic a) $CsPbBr_3$ and b) $CsSrBr_3$. c) Normalized interatomic force constants as a function of the interatomic distance for $CsPbBr_3$ (red circles) and $CsSrBr_3$ (blue triangles). The filled markers show the M–Br force constants and the unfilled markers show all other interactions.

Finally, in Supplementary Figure 12 we show the perturbation to the charge density upon a single Br displacement along the M−Br−M axis. The impact of the LPE and the corresponding resonant bonding is evident from the long-range perturbation to the charge density in $CsPbBr_3$ (Figure 12a). Resonance is reduced in $CsSrBr_3$ due to the reduced covalency and homopolar bonding fraction [38, 39], but is not completely removed (see Supplementary Figure 12b). The disorder potential, calculated using the method described in Ref 29, also shortens dynamically in $CsSrBr_3$ due to the Br motions. The transversal Br motion is energetically more favorable, as we have previously discussed,[40] and facilitates octahedral rotations. This feature of $CsPbBr_3$ is also present in $CsSrBr_3$ (see Supplementary Figures 12c and d).

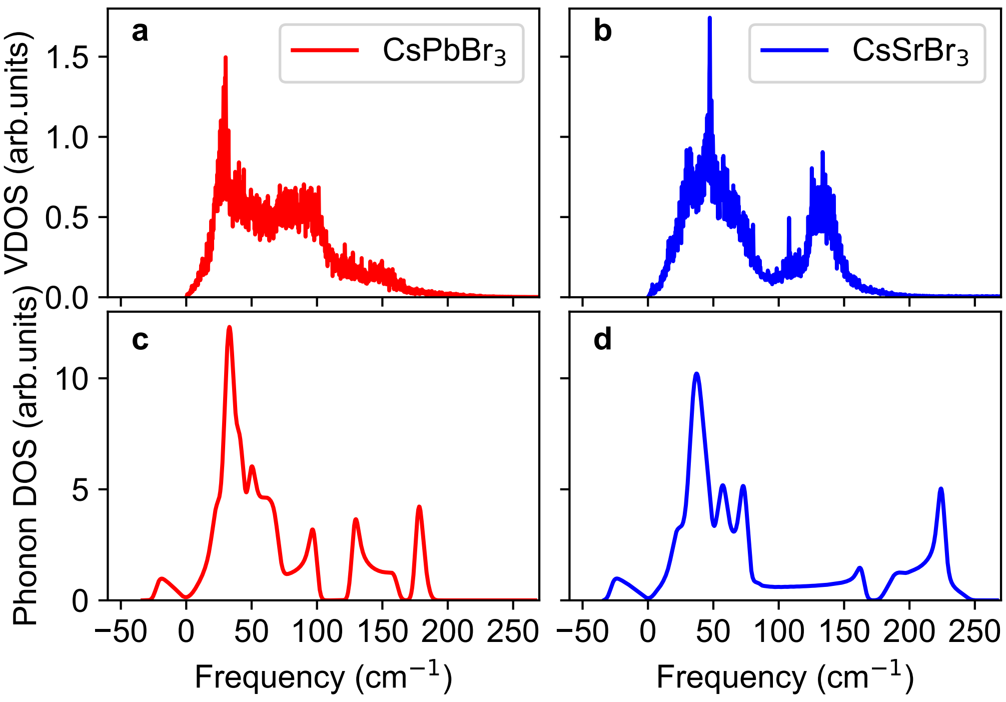

Supplementary Figure 10. **Vibrational density of states.** VDOS calculated as the power spectrum of the VACF from MD trajectories of cubic a) $CsPbBr_3$ at 525 K and b) $CsSrBr_3$ at 570 K. The phonon DOS of the cubic phase calculated using finite differences is shown in panels c) and d). Notice the finite phonon DOS at negative frequencies, which indicate imaginary phonon modes.

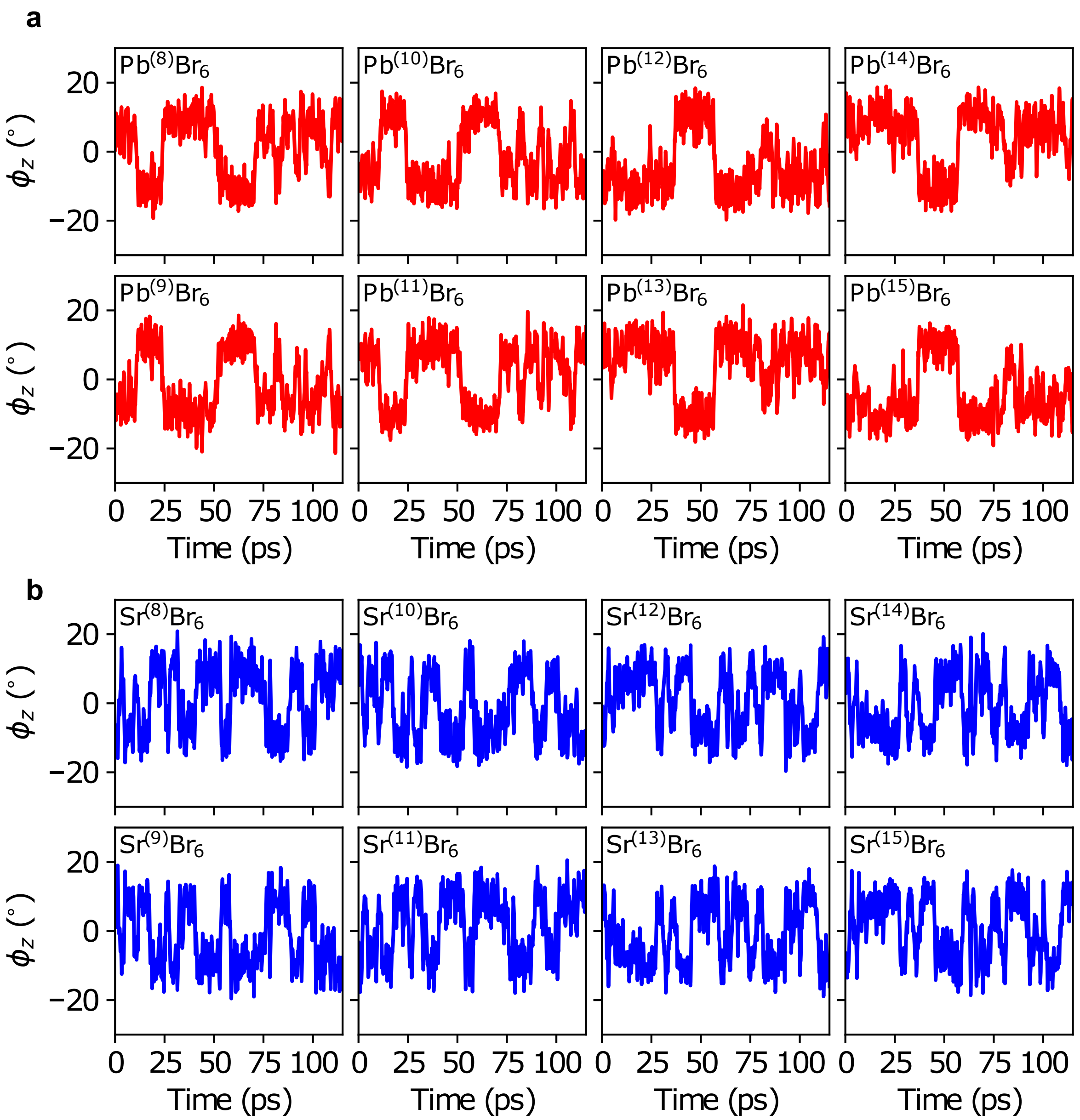

Supplementary Figure 11. **Time evolution of rotation angles.** Rotation angle around z, ϕ_z, of individual octahedra (denoted as $M^{(i)}$, with i the M-site index) in the supercell as a function of time from DFT-based MD simulations of cubic a) $CsPbBr_3$ and b) $CsSrBr_3$.

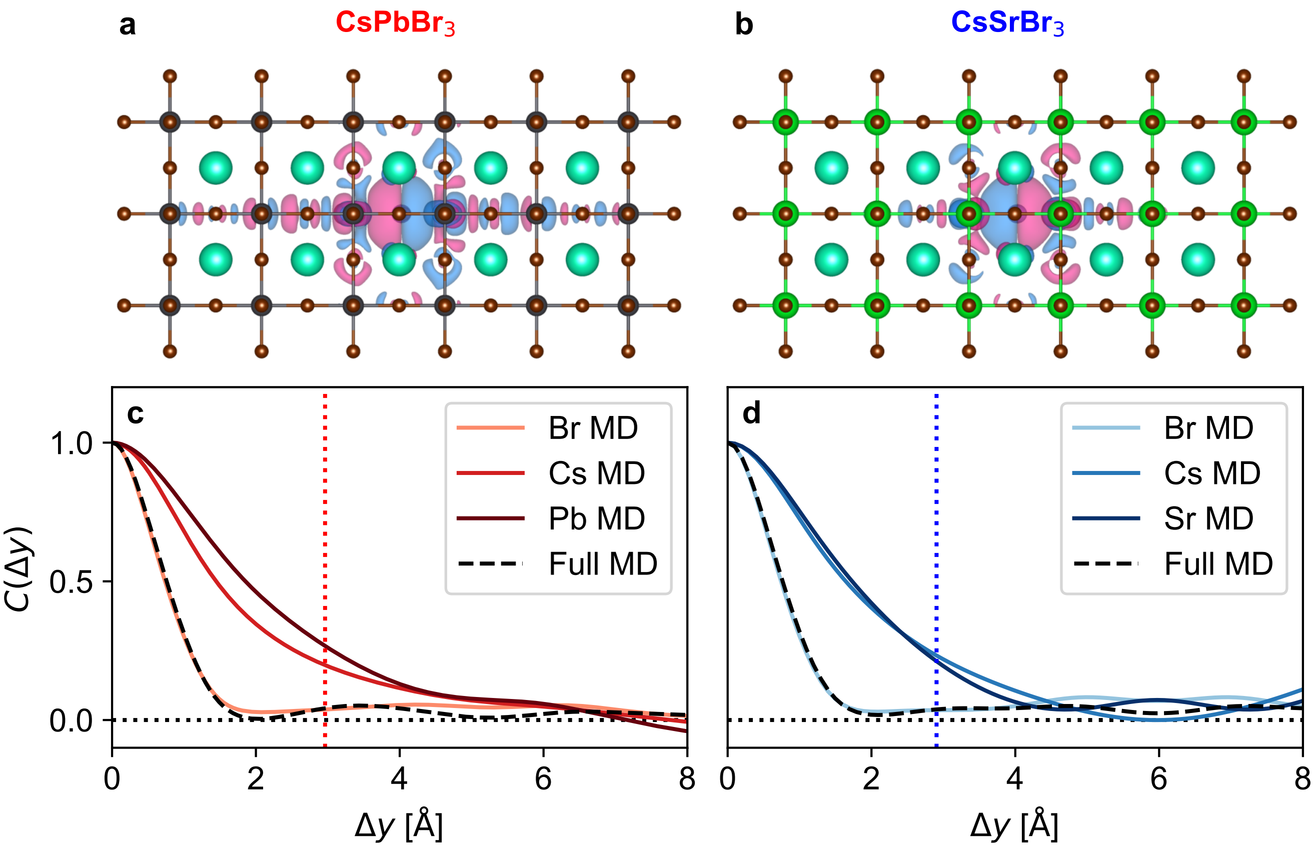

Supplementary Figure 12. **Shortening of disorder correlation.** Isosurface representations of the changes in the charge density ($\geq 8.1 \times 10^{-3}$ eÅ^{-3}) upon Br displacement along the Br−M−Br bonding direction (5% of lattice constant) for a) $CsPbBr_3$ and b) $CsSrBr_3$. The autocorrelation of disorder potentials, $C(\Delta y)$, calculated for the full MD trajectories, as well as the contribution of the individual species (for details Ref. 29), for $CsPbBr_3$ and $CsSrBr_3$, are shown in panels c) and d), respectively.

Supplementary References

[1] Bersuker, I. *The Jahn-Teller Effect* (Cambridge University Press, 2006).
[2] Fabini, D. H. *et al.* Noncollinear electric dipoles in a polar, chiral phase of $CsSnBr_3$: Existence and limits of bulk rashba effects in perovskite halides (2024). arXiv:2401.07978.
[3] Bersuker, I. On the origin of ferroelectricity in perovskite-type crystals. *Phys. Lett.* **20**, 589–590 (1966).
[4] Van der Vorst, C. & Maaskant, W. Stereochemically active $(5s)^2$ lone pairs in the structures of α-InCl and β-InCl. *J. Solid State Chem.* **34**, 301–313 (1980).
[5] Du, M.-H. & Singh, D. J. Enhanced Born charge and proximity to ferroelectricity in thallium halides. *Phys. Rev. B* **81**, 144114 (2010).
[6] Fabini, D. H. *et al.* Dynamic Stereochemical Activity of the Sn^{2+} Lone Pair in Perovskite $CsSnBr_3$. *J. Am. Chem. Soc.* **138**, 11820–11832 (2016).
[7] Gao, L. *et al.* Metal cation s lone-pairs increase octahedral tilting instabilities in halide perovskites. *Mater. Adv.* **2**, 4610–4616 (2021).
[8] Ok, K. M. *et al.* Distortions in octahedrally coordinated d^0 transition metal oxides: A continuous symmetry measures approach. *Chem. Mater.* **18**, 3176–3183 (2006).
[9] Waghmare, U. V., Spaldin, N. A., Kandpal, H. C. & Seshadri, R. First-principles indicators of metallicity and cation off-centricity in the IV-VI rocksalt chalcogenides of divalent Ge, Sn, and Pb. *Phys. Rev. B* **67** (2003).
[10] Birch, F. Finite Elastic Strain of Cubic Crystals. *Phys. Rev.* **71**, 809–824 (1947).
[11] Murnaghan, F. D. The Compressibility of Media under Extreme Pressures. *Proc. Natl. Acad. Sci. U.S.A.* **30**, 244–247 (1944).
[12] Loyd, M. *et al.* Crystal structure and thermal expansion of $CsCaI_3$:Eu and $CsSrBr_3$:Eu scintillators. *J. Cryst. Growth* **481**, 35–39 (2018).
[13] Berreman, D. W. & Unterwald, F. C. Adjusting Poles and Zeros of Dielectric Dispersion to Fit Reststrahlen of $PrCl_3$ and $LaCl_3$. *Phys. Rev.* **174**, 791–799 (1968).
[14] Servoin, J. L., Luspin, Y. & Gervais, F. Infrared dispersion in $SrTiO_3$ at high temperature. *Phys. Rev. B* **22**, 5501–5506 (1980).
[15] Ostapchuk, T. *et al.* Polar phonons and central mode in antiferroelectric $PbZrO_3$ ceramics. *J. Phys.: Condens. Matter* **13**, 2677–2689 (2001).
[16] Moreira, R. L. *et al.* Microwave and infrared dielectric properties of $Sr_{1\text{-}3x/2}Ce_xTiO_3$($x$=0.154–0.400) incipient ferroelectrics at cryogenic temperatures. *J. Phys. D: Appl. Phys.* **42**, 075411 (2009).
[17] Sendner, M. *et al.* Optical phonons in methylammonium lead halide perovskites and implications for charge transport. *Mater. Horiz.* **3**, 613–620 (2016).
[18] Yu, P. Y. & Cardona, M. *Fundamentals of semiconductors* (Springer, 2010).
[19] Berreman, D. W. Infrared Absorption at Longitudinal Optic Frequency in Cubic Crystal Films. *Phys. Rev.* **130**, 2193–2198 (1963).
[20] Gervais, F. & Piriou, B. Anharmonicity in several-polar-mode crystals: adjusting phonon self-energy of LO and TO modes in Al_2O_3 and TiO_2 to fit infrared reflectivity. *J. Phys. C: Solid State Phys.* **7**, 2374–2386 (1974).
[21] Skelton, J. M. *et al.* Lattice dynamics of the tin sulphides SnS_2, SnS and Sn_2S_3: vibrational spectra and thermal transport. *Phys. Chem. Chem. Phys.* **19**, 12452–12465 (2017).
[22] Skelton, J. M. Phonopy-Spectroscopy. URL https://github.com/skelton-group/Phonopy-Spectroscopy/.
[23] Porezag, D. & Pederson, M. R. Infrared intensities and Raman-scattering activities within density-functional theory. *Phys. Rev. B* **54**, 7830–7836 (1996).
[24] Mitroy, J., Safronova, M. S. & Clark, C. W. Theory and applications of atomic and ionic polarizabilities. *J. Phys. B: At. Mol. Opt. Phys.* **43**, 202001 (2010).
[25] Gajdoš, M., Hummer, K., Kresse, G., Furthmüller, J. & Bechstedt, F. Linear optical properties in the projector-augmented wave methodology. *Phys. Rev. B* **73**, 1–9 (2006).
[26] Dove, M. T. *Introduction to Lattice Dynamics*. Cambridge Topics in Mineral Physics and Chemistry (Cambridge University Press, Cambridge, 1993).
[27] Togo, A. & Tanaka, I. First principles phonon calculations in materials science. *Scripta Materialia* **108**, 1–5 (2015).
[28] Zhu, T. & Ertekin, E. Mixed phononic and non-phononic transport in hybrid lead halide perovskites: Glass-crystal duality, dynamical disorder, and anharmonicity. *Energy Environ. Sci.* **12**, 216–229 (2019).
[29] Gehrmann, C. & Egger, D. A. Dynamic shortening of disorder potentials in anharmonic halide perovskites. *Nat. Commun.* **10**, 3141 (2019).
[30] Lee, S. *et al.* Resonant bonding leads to low lattice thermal conductivity. *Nat. Commun.* **5**, 1–8 (2014).
[31] Lee, C., Vanderbilt, D., Laasonen, K., Car, R. & Parrinello, M. *Ab initio* studies on the structural and dynamical properties of ice. *Phys. Rev. B* **47**, 4863–4872 (1993).
[32] Meshkov, S. V. Low-frequency dynamics of Lennard-Jones glasses. *Phys. Rev. B* **55**, 12113–12120 (1997).
[33] Flores Ruiz, H. M. *Modos vibracionales de baja frecuencia y su impacto en la formación de vidrios*. Ph.D. thesis, Universidad Nacional Autónoma de México, México D.F. (2012).
[34] Nielsen, M. D., Ozolins, V. & Heremans, J. P. Lone pair electrons minimize lattice thermal conductivity. *Energy Environ. Sci.* **6**, 570–578 (2013).
[35] Skelton, J. M. *et al.* Anharmonicity in the High-Temperature *Cmcm* Phase of SnSe: Soft Modes and Three-Phonon

Interactions. *Phys. Rev. Lett.* **117**, 075502 (2016).
[36] Chang, C. & Zhao, L. D. Anharmoncity and low thermal conductivity in thermoelectrics. *Mater. Today Phys.* **4**, 50–57 (2018).
[37] Fransson, E. *et al.* Limits of the phonon quasi-particle picture at the cubic-to-tetragonal phase transition in halide perovskites. *Commun. Phys.* **6**, 173 (2023).
[38] Krebs, H. Der EinfluB homoopolarer Bindungsanteile auf die Struktur anorganischer Salze. *Z. Für Anorg. Chem.* **278**, 82 (1955).
[39] Krebs, H. Der Einfluss homöopolarer Bindungsanteile auf die Struktur anorganischer Salze. II. Halbleiter und legierungsartige Phasen. *Acta Crystallogr.* **9**, 95–108 (1956).
[40] Gehrmann, C., Caicedo-Dávila, S., Zhu, X. & Egger, D. A. Transversal Halide Motion Intensifies Band-To-Band Transitions in Halide Perovskites. *Adv. Sci.* **9**, 2200706 (2022).
